# Diuretic Action of Apelin-13 Mediated by Inhibiting cAMP/PKA/sPRR Pathway

**DOI:** 10.3389/fphys.2021.642274

**Published:** 2021-03-31

**Authors:** Yanting Chen, Chuanming Xu, Jiajia Hu, Mokan Deng, Qixiang Qiu, Shiqi Mo, Yanhua Du, Tianxin Yang

**Affiliations:** ^1^Zhongshan School of Medicine, Institute of Hypertension, Sun Yat-sen University, Guangzhou, China; ^2^Center for Translational Medicine, Jiangxi University of Traditional Chinese, Nanchang, China; ^3^Department of Pharmacology, Sun Yat-sen University School of Medicine, Guangzhou, China; ^4^Department of Internal Medicine, University of Utah and Veterans Affairs Medical Center, Salt Lake City, UT, United States

**Keywords:** soluble prorenin receptor, apelin, AQP2, urinary concentration, water dehydration

## Abstract

Emerging evidence is showing that apelin plays an important role in regulating salt and water balance by counteracting the antidiuretic action of vasopressin (AVP). However, the underlying mechanism remains unknown. Here, we hypothesized that (pro) renin receptor (PRR)/soluble prorenin receptor (sPRR) might mediate the diuretic action of apelin in the distal nephron. During water deprivation (WD), the urine concentrating capability was impaired by an apelin peptide, apelin-13, accompanied by the suppression of the protein expression of aquaporin 2 (AQP2), NKCC2, PRR/sPRR, renin and nuclear β-catenin levels in the kidney. The upregulated expression of AQP2 or PRR/sPRR both induced by AVP and 8-Br-cAMP was blocked by apelin-13, PKA inhibitor (H89), or β-catenin inhibitor (ICG001). Interestingly, the blockage of apelin-13 on AVP-induced AQP2 protein expression was reversed by exogenous sPRR. Together, the present study has defined the cyclic adenosine monophosphate (cAMP)/protein kinase A (PKA)/sPRR pathway in the CD as the molecular target of the diuretic action of apelin.

## Introduction

The kidney plays a critical role in the maintenance of salt and water balance. It is well established that antidiuretic action of vasopressin (AVP) binds to its V2 receptor (V2R) in the basolateral membrane of collecting duct (CD) to activate the cyclic adenosine monophosphate (cAMP)/protein kinase A (PKA) pathway. When activated, this pathway phosphorylates aquaporin 2 (AQP2) to induce its trafficking to the apical membrane in the CD principal cells, leading to increased membrane permeability and water reabsorption (Nielsen et al., 2002). Apelin, an endogenous vasoactive ligand of the apelin receptor [Aplnr, Apelin peptide jejunum (APJ)], (Tatemoto et al., 1998), plays a key role in regulating salt and water balance by counteracting the antidiuretic action of AVP (Hus-Citharel et al., 2014). There are three active biological forms of apelin, including apelin-13 (amino acid residues 65–77), apelin-17 (amino acid residues 61–77), and apelin-36 (amino acid residues 42–77), with apelin-13 being the most active isoform.

The production of apelin is under the control of plasma osmolality. Increased plasma osmolality decreased (De Mota et al., 2004; Azizi et al., 2008), whereas reduced plasma osmolality increased (Azizi et al., 2008) plasma apelin levels. Additionally, levels of apelin were altered in many disease models. For example, Zhang et al. (2020) reported that *Apln* mRNA level increases both in the renal cortex and medulla in diabetic rats with renal ischemia-reperfusion injury, and apelin expression was significantly downregulated in pulmonary arterial hypertension and heart failure (Yang et al., 2015). We previously reported that renal *Apln* mRNA expression was controlled by (pro)renin receptor (PRR) and significantly downregulated in high-salt-fed Dahl salt-sensitive rats (Xu et al., 2020). Recently, Hus-Citharel et al. (2014) first reported that the diuretic action of apelin-17 is mediated by inhibiting central AVP release as well as its peripheral action on AQP2 trafficking in the CD. As an extension of this study, Boulkeroua et al. (2019) recently reported that apelin-13 suppressed AVP-induced cAMP production and AQP2 expression and trafficking in cultured mpkCCD cells. However, the exact mechanisms involved in these actions of apelin remains elusive.

(Pro)renin receptor, a new component of the renin-angiotensin system (RAS), acts as a specific receptor for prorenin and renin to regulate their catalytic activity (Nguyen et al., 2002) and a regulator of intrarenal RAS activity (Yang and Chuanming, 2017). PRR plays an essential role in determining renal AQP2 expression and urine concentrating capability as established by compelling evidence from pharmacologic and conditional gene knockout studies (Ramkumar et al., 2015; Trepiccione et al., 2016; Wang et al., 2016). AVP-stimulated AQP2 expression depends on prorenin activation of PRR (Wang et al., 2016), conditional deletion of PRR in the CD, and the whole nephron causes a common polyuria phenotype with consistent inhibition of renal AQP2 and V2R expression and AVP sensitivity (Ramkumar et al., 2015; Trepiccione et al., 2016; Wang et al., 2016). PRR is cleaved by a protease to generate a 28-kDa soluble prorenin receptor (sPRR), and among multiple candidate proteases, site-1 protease (S1P) represents the predominant enzymatic source of sPRR (Fang et al., 2017; Nakagawa et al., 2017). We recently reported that S1P-derived sPRR exerts a biological action in the regulation of renal AQP2 and V2R expression *via* the β-catenin pathway, and thus enhances urine concentrating capability (Lu et al., 2016; Wang et al., 2019). The goal of the present study was to examine the possible interaction between apelin and PRR in the setting of enhanced AVP action such as WD.

## Materials and Methods

### Animals Care

Male C57BL/6 mice (2-month old) were purchased from Beijing Charles River Laboratories (Beijing, China). All animals were housed in a temperature- and humidity-controlled room with a 12:12-h light–dark cycle, given free access to tap water, and fed the standard diet. The animal protocols were approved by the Animal Care and Use Committee at Sun Yat-sen University, China.

### Water Deprivation

The mice were divided into three groups (control, WD, and WD + apelin-13 groups). Mice were housed in metabolic cages for 7 days in a temperature- and humidity-controlled room with a 12:12-h light–dark cycle for 24-h urine collection. Control and WD mice received vehicle injection, WD + apelin-13 mice received apelin-13 (Purity ≥ 95%, 057-18, Phoenix pharmaceuticals) injection by the intraperitoneal route (100 μg kg^−1^·day^−1^, three times a day for every 8 h) for 7 days. In the first 5 days, all mice were given free access to tap water and chow diets. In the last 2 days, control mice were given free access to tap water and chow diets, WD and WD + apelin-13 mice were deprived of water for 48 h but had free access to chow diets. Urine samples were collected every 24 h. At the end of the experiment, mice were euthanized, and blood and kidneys were harvested. The osmolality of urine and serum was determined by using freezing-point depression (OM 806, Osmometer, Loser, Germany).

### Chronic Water Loading (WL)

All mice were placed in metabolic cages and fed a normal chow diet and tap water. After 24-h urine collection at baseline, the mice were randomly divided into control or water loading (WL) group. Mice in the control group were fed the normal chow diet, and the mice in WL group received high water-content food (72% water content) for 7 days. The gelled diets were made by melting agar (1% by weight) in water (72%), cooling, and adding the ground chow (27%) and NaCl (0.5%). The final content of NaCl became 0.8%. On day 7, daily urine output was collected, and mice were euthanized to harvest blood and kidneys.

### Enzyme Immunoassay

To detect medium sPRR and cAMP from primary inner medullary collecting duct (IMCD) cells (Control, AVP, and AVP + Apelin-13 groups), and urinary apelin levels in WD (Control and WD groups) and WL (Control and WL groups) mice, we used the following commercially available enzyme immunoassay kits according to the manufacturer’s instructions: sPRR (27782, Immuno-Biological Laboratories), cAMP (581001, Cayman Chemical), and apelin (CED066Mu, Cloud-Clone Corp), respectively.

### Immunoblotting

Tissue samples from the renal cortex and medulla, and cell samples, were lysed and subsequently sonicated in RIPA buffer (Biocolors, Shanghai, China) with protease inhibitor cocktail (Roche, Berlin, Germany). Protein concentrations were determined with the Pierce BCA Protein Assay Kit (Cat# NCI3225CH, Thermo Scientific, Rockford, IL, United States) according to the manufacturer’s instructions. About 30 mg of protein for each sample were denatured in boiling water bath for 5 min, then resolved by SDS-PAGE and transferred onto polyvinylidene fluoride membrane (Immobilion-P, Millipore, Bedford, MA, United States). The membranes were blocked with 10% nonfat dry milk in Tris-buffered saline with Tween-20 (TBST) for 1 h at room temperature, followed by incubation with primary antibodies [AQP2, 1:1000 dilution, A7310, Sigma-Aldrich; Na/K/2Cl cotransporter (NKCC2), 1:1000 dilution, SAB5200103, Sigma-Aldrich; PRR, 1:1000 dilution, HPA003156, Sigma-Aldrich; Renin (B-12), 1:300 dilution, sc-133145, Santa Cruz; ACE, 1:1000 dilution, ab39172, Abcam; Phospho-CREB (Ser133), 1:1000 dilution, 9198, Cell Signaling Technology; CREB (48H2), 1:1000 dilution, 9197, Cell Signaling Technology; β-catenin, 1:1000 dilution, ARG52651, Arigo; α-Na^+^-K^+^-ATPase (α-NKA), 1:1000 dilution, ab76020, Abcam; β-actin, 1:10,000 dilution, A-2066, Sigma-Aldrich] diluted in antibody dilution buffer [1.5 g bovine serum albumin (BSA), 0.1 g NaN_3_, 50 ml TBST] overnight at 4°C. After being washed with TBST, membranes were incubated with secondary antibodies [goat anti-rabbit/mouse horseradish peroxidase (HRP) – conjugated secondary antibody] (Thermo Scientific) for 1 h at room temperature and visualized with enhanced chemiluminescence (Thermo Scientific). Signals on immunoblots were detected using Tanon 5200 Luminescent Imaging Workstation (Tanon, Shanghai, China) and quantitated using the accompanying densitometry software (UVP, Upland, CA, United States). The expression of the protein levels was corrected to β-actin.

### Cell Membrane, Cytoplasmic, and Nuclear Protein Fraction Isolation

The membrane, cytosolic, and nuclear fractions of proteins were extracted using a kit according to the manufacturer’s instructions (KGBSP002, KeyGen Biotech, Nanjing, China).

### Quantitative Reverse Transcriptase PCR

Total RNA was extracted according to the manufacturer’s instructions for TRIzol reagent (Invitrogen). We used 1.2 μg of total RNA for reverse transcription by using the Transcriptor First Strand cDNA Synthesis Kit (04379012001, Roche). Quantitative PCR (qPCR) was performed using the ABI Prism StepOnePlus System (Applied Biosystems) and the SYBR Premix Ex Taq kit (Tli RNaseH Plus; Roche) according to the manufacturer’s instructions. Primers are as follows: for *PRR*, 5'-TCTCCGAACTGCAAGTGCTA-3' (sense) and 5'-CTGCAAACTTTTGGAGAGCA-3' (antisense); for *AQP2*, 5'-GGACCTGGCTGTCAATGCT-3' (sense) and 5'-ATCGGTGGAGGCAAAGATG-3' (antisense); for *NKCC2*, 5'-GCTCTTCATTCGCCTCTCCT-3' (sense) and 5'-AGCCTATTGACCCACCGAAC-3' (antisense); for *renin 1*, 5'-CTCTCTGGGCACTCTTGTTGC-3' (sense) and 5'-GGGAGGTAAGATTGGTCAAGGA-3' (antisense); for *ACE*, 5'-TTGCTATGGGCATGGAAGAG-3' (sense) and 5'-CAGGTCTTGCTCCAGGTTGT-3' (antisense); for *Apela*, 5'-TTTGCAGAGACTTCCCGCTT-3' (sense) and 5'-GCTCACCCCACATCCTATGG-3' (antisense); for *Apln*, 5'- GCTGCTGCTGCTCTGGCTCT-3' (sense) and 5'-GGGGGCGCTGTCTGCGAAAT-3' (antisense); for *Aplnr*, 5'-GCCTGTCATGGTGTTCCG-3' (sense) and 5'- CTCAATGCGCTCCTTTCGG-3' (antisense); for *GAPDH*, 5'-AGGTCGGTGTGAACGGATTTG-3' (sense) and 5'-TGTAGACCATGTAGTTGAGGTCA-3' (antisense). All reactions were run in duplicate. The data was shown as a relative value normalized by *GAPDH*.

### Primary Cultures of Rat Inner Medullary Collecting Duct (IMCD) Cells

Primary cultures of rat renal IMCD cells were prepared from pathogen-free male Sprague-Dawley (SD) rats (4-week-old) in hypertonic conditions, as previously described (Faust et al., 2013; Xu et al., 2016). The protocols were approved by the Institutional Animal Care and Use Committee of Sun Yat-sen University, China. The IMCD cells were grown in six-well plates with hypertonic medium [Dulbecco’s modified Eagle’s medium (DMEM)-Ham’s F-12 medium containing 10% (vol/vol) fetal bovine serum, 0.5 μM 8-Br-cAMP, 130 mM sodium chloride, and 80 mM urea]. Upon confluence, cells were serum-starved for 12 h, and then pretreated with 100 nM apelin-13 for 2 h. We then treated the cells with 10 nM AVP or 10 μM forskolin (FSK) /100 μM 3-Isobutyl-1-methylxanthine (IBMX) for 24 h. In an independent experiment, cells were pretreated with 10 μM PKA inhibitor H89, and then incubated with 100 μM cAMP analog 8-Br-cAMP (ab141448, Abcam) for 24 h. Separately, cells were pretreated with 10 μM H89 for 1 h, then treated with 10 nM sPRR-His for 2 h followed by 24-h treatment with 10 nM AVP. In another *in vitro* study, we pretreated the cells with 10 μM ICG001, and then incubated them with 10 nM AVP or 100 μM 8-Br-cAMP for 24-h. At the end of all the experiments, the cells were harvested for PRR and AQP2 expression analysis. The cell medium was collected for sPRR and cAMP assay by using a soluble (Pro) renin Receptor Assay kit (27782, Immuno-Biological Laboratories, Gunma, Japan) and cAMP EIA kit (581001, Cayman Chemical). According to the manufacturer’s instructions, and the values were calculated and corrected to cellular protein content.

### Statistical Analysis

Data are summarized as means ± SE. Statistical analysis was performed using ANOVA with the Bonferroni test for multiple comparisons or unpaired Student’s *t*-test for two comparisons. *p* < 0.05 was considered statistically significant.

## Results

### Regulation of Renal Apelinergic System Components During WD and WL

**Figure 1:**
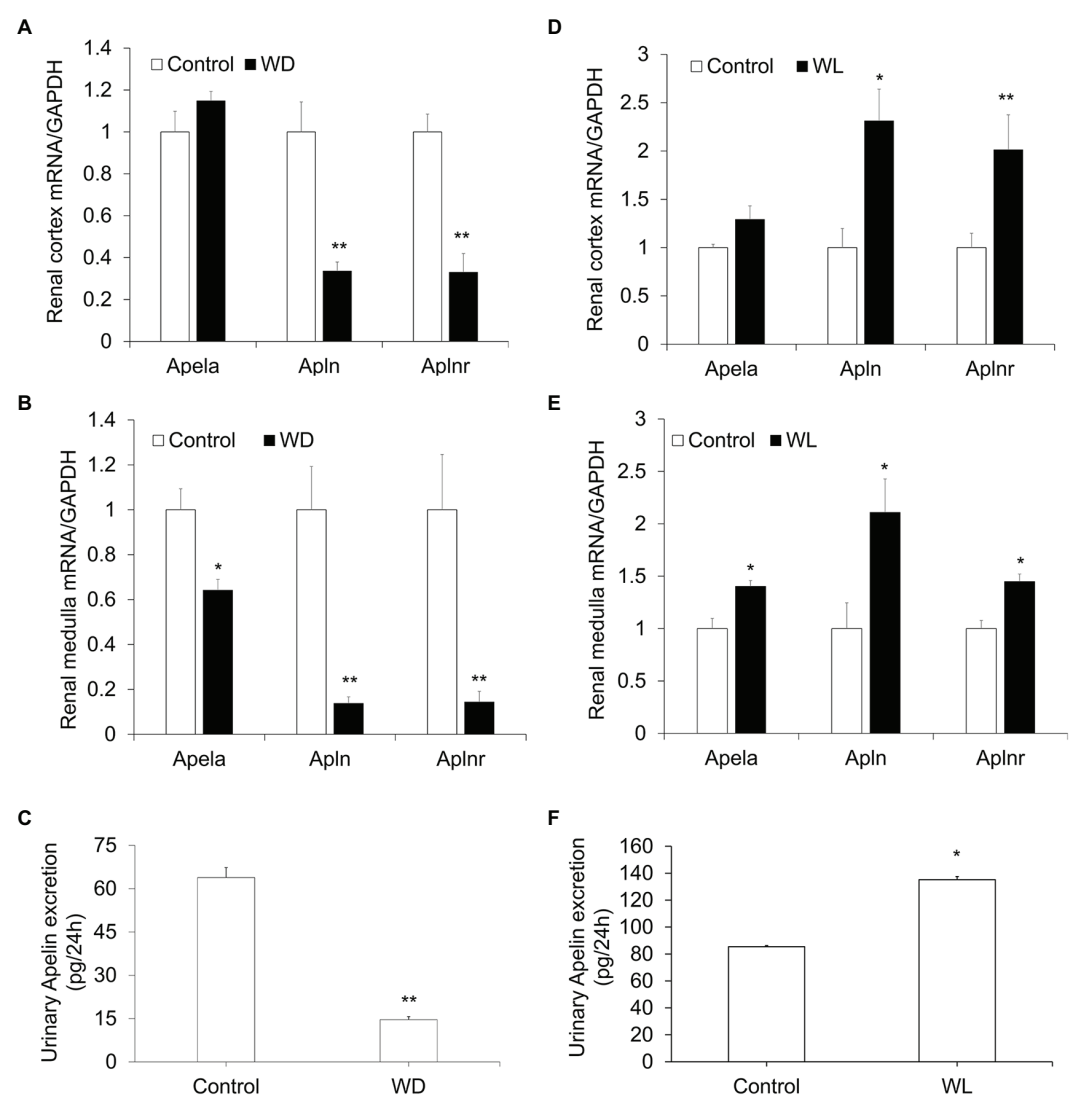
The response of the renal apelinergic system to water deprivation (WD) and chronic water loading (WL) in mice. **(A,B)** Quantitative reverse transcriptase PCR (qRT-PCR) analysis of renal *Apln*, *Apela*, and *Aplnr* mRNA expression in the kidney cortex **(A)** and medulla **(B)** in WD mice, expression was normalized by *GAPDH*. **(C)** A 24-h urinary apelin excretion in WD mice. **(D,E)** qRT-PCR analysis of renal *Apln*, *Apela*, and *Aplnr* mRNA expression in kidney cortex **(D)** and medulla **(E)** in chronic WL mice. The expression was normalized by *GAPDH*. **(F)** 24-h urinary apelin excretion in WL mice. *n* = 5 per group. Data are mean ± SEM. ^*^p < 0.05 and ^**^p < 0.01 vs. Control.

Although several previous studies have indicated the diuretic action of apela (Deng et al., 2015), APJ (Roberts et al., 2009), and apelin (Hus-Citharel et al., 2014), the underlying mechanism largely remains unknown. To assess whether the renal apelinergic system was involved in regulation of water homeostasis, the mRNA level of *Apela* (encoding ELABELA, ELA), *Apln* (encoding apelin), and *Aplnr* (encoding APJ), as well as 24-h urinary apelin excretion, were examined in WD and WL mice. As shown in Figure 1, WD markedly decreased (Figures 1A–C) whereas WL significantly increased (Figures 1D–F), renal *Apela*, *Apln*, and *Aplnr* mRNA levels as assessed by quantitative reverse transcriptase PCR (qRT-PCR), and 24-h urinary apelin excretion as assessed by ELISA. These results are compatible with the generally diuretic action of renal apelinergic system.

### Apelin-13 Inhibits WD-Induced Renal AQP2 Expression and Trafficking

**Figure 2:**
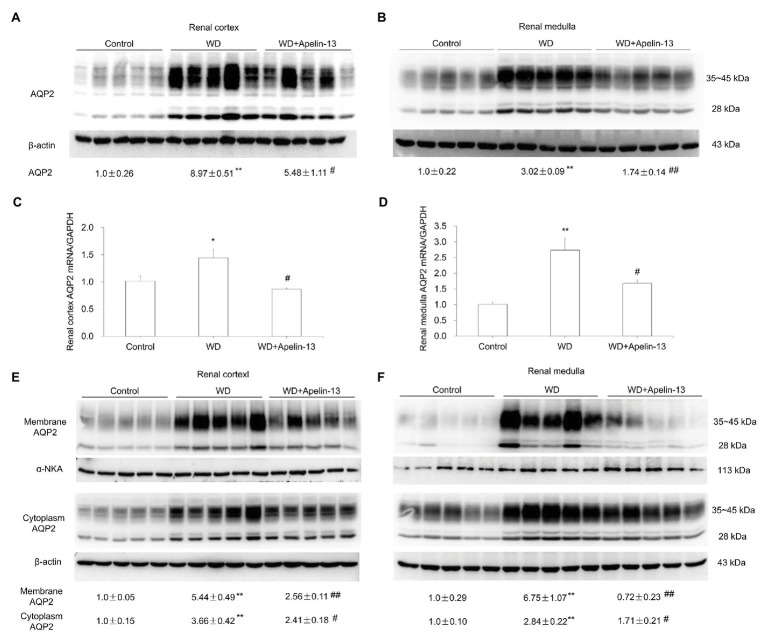
Effect of apelin-13 on aquaporin 2 (AQP2) expression in the renal cortex and medulla in WD mice. **(A,B)** Representative immunoblotting and densitometric analysis of AQP2 protein expression in the kidney cortex **(A)** and medulla **(B)**, the protein abundances were normalized by β-actin **(C,D)** qRT-PCR analysis of renal *AQP2* mRNA expression in the kidney cortex **(C)** and medulla **(D)**. The expression was normalized by *GAPDH*. **(E,F)** Representative immunoblotting and densitometric analysis of membrane and cytosolic fraction AQP2 protein expression in the kidney cortex **(E)** and medulla **(F)**. The abundance of AQP2 in the membranous and cytoplasmic factions was normalized by α-NKA and β-actin, respectively. *n* = 5 per group. Data are mean ± SEM. ^*^p < 0.05 and ^**^p < 0.01 vs. Control; ^#^*p* < 0.05 and ^##^*p* < 0.01 vs. WD.

We examined the functional role of apelin in urine concentration by using apelin-13, the highest biological active form of apelin. The 48-h WD treatment decreased urine volume and increased both urine and plasma osmolality in the vehicle group. Apelin-13 infusion partially attenuated the rise in urine osmolality and further increased plasma osmolality during WD (Table 1). By immunoblotting, renal AQP2 was detected as a 28-kD band (nonglycosylated) and 35- to 45-kD bands (glycosylated). AQP2 protein abundance (Figures 2A,B), as well as AQP2 mRNA levels (Figures 2C,D) in both the cortex and medulla were elevated after WD in the vehicle group, and this increase was less in apelin-13-treated animals.

**Table 1:** General physiological data in C57BL/6 mice.

		Food intake (g/24h)	Urine volume (ml/24 h)	Urine osmolality (mosmol/kg·H_2_O)	Plasma osmolality (mosmol/kg·H_2_O)	Δ Body weight (g)
Control		4.50 ± 0.09	1.27 ± 0.18	1426.67 ± 76.98	315.67 ± 1.23	0.31 ± 0.33
WD	24 h	2.82 ± 0.04^**^	0.96 ± 0.06	3051.67 ± 67.30^**^		-0.03 ± 0.31
	48 h	1.96 ± 0.09^**^	0.48 ± 0.10^**^	3711.67 ± 72.70^***^	328.20 ± 1.72^***^	-1.37 ± 0.26^**^
WD + Apelin-13	24 h	2.83 ± 0.04^**^	1.06 ± 0.07	2945.00 ± 50.32^**^		-0.40 ± 0.21^*^
	48 h	1.92 ± 0.03^**^	0.47 ± 0.08^**^	3398.33 ± 60.11^***^^#^	335.80 ± 2.98^***^^#^	-1.65 ± 0.23^**^

* *p* < 0.05;

** *p* < 0.01;

*** *p* < 0.001 vs. Control;

# *p* < 0.05 vs. WD-48h.

The antidiuretic action of AVP mainly depends on V2R-mediated action on AQP2 trafficking acutely by increasing phosphorylation of AQP2 and chronically by stimulating AQP2 transcription in the CD (Nielsen et al., 2002). Here, the trafficking event was evaluated by examining the abundance of AQP2 protein in the fractionated tissue samples. Consistent with the *in vitro* observation by Boulkeroua et al. (2019) that apelin-13 antagonized the hydroosmotic effect of AVP on AQP2 expression and trafficking in cultured mpkCCD cells, we found that AQP2 abundance in both cytosolic and membranous fractions in both renal cortex (Figure 2E) and medulla (Figure 2F) were elevated by WD which was blocked by apelin-13 treatment. These results suggest that apelin may regulate not only AQP2 gene transcription but also AQP2 trafficking.

### Apelin-13 Suppresses WD-Induced Renal NKCC2 Expression

**Figure 3:**
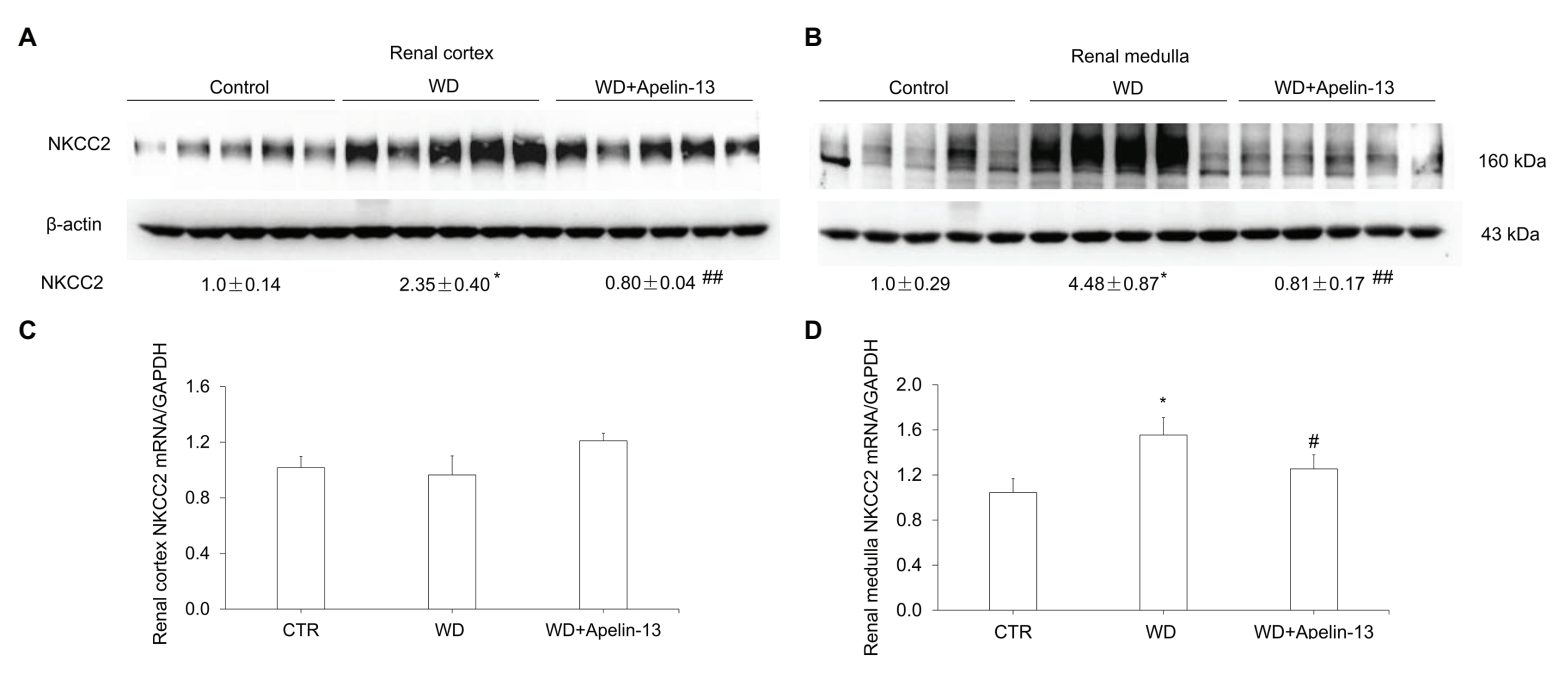
Effect of apelin-13 on NKCC2 expression in the renal cortex and medulla in WD mice. **(A,B)** Representative immunoblotting and densitometric analysis of NKCC2 protein expression in the kidney cortex **(A)** and medulla **(B)**, the protein abundances were normalized by β-actin. **(C,D)** qRT-PCR analysis of renal *NKCC2* mRNA expression in the kidney cortex **(C)** and medulla **(D)**, expression was normalized by *GAPDH*. *n* = 4–5 per group. Data are mean ± SEM. ^*^p < 0.05 and ^**^p < 0.01 vs. Control; ^#^*p* < 0.05 and ^##^*p* < 0.01 vs. WD.

Besides the CD, the thick ascending limb (TAL) is another important nephron site for the antidiuretic action of AVP (Bachmann and Mutig, 2017). Total protein abundance of NKCC2 in both cortex (Figure 3A) and medulla (Figure 3B), and NKCC2 mRNA levels in the medulla (Figure 3D) but not cortex (Figure 3C), were elevated after WD in the vehicle group, which were completely abolished by apelin-13 treatment.

### Apelin-13 Attenuates WD-Induced Activation of Intrarenal RAS

**Figure 4:**
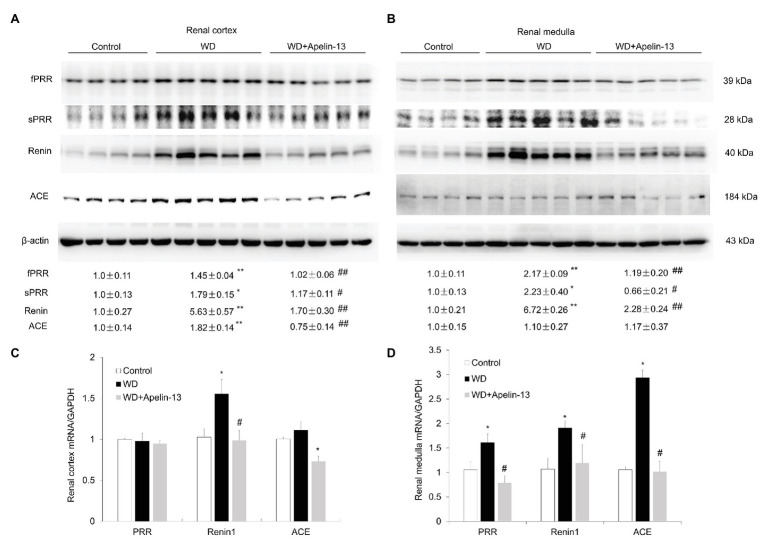
Effect of apelin-13 on intrarenal renin-angiotensin system (RAS) in the renal cortex and medulla in WD mice. **(A,B)** Representative immunoblotting and densitometric analysis of fPRR, soluble prorenin receptor (sPRR), renin, and ACE protein expression in the kidney cortex **(A)** and medulla **(B)**, the protein abundances were normalized by β-actin. **(C,D)** qRT-PCR analysis of renal *PRR*, *renin 1*, and *ACE* mRNA expression in the kidney cortex **(C)** and medulla **(D)**, expression was normalized by *GAPDH*. *n* = 4–5 per group. Data are mean ± SEM. ^*^p < 0.05 and ^**^p < 0.01 vs. Control; ^#^*p* < 0.05 and ^##^*p* < 0.01 vs. WD.

Emerging evidence has demonstrated that intrarenal RAS is involved in the regulation of urine concentrating capability (Li et al., 2012). We found that the expression of PRR/sPRR and renin protein in the kidney cortex (Figures 4A,C) and medulla (Figures 4B,D) was significantly elevated after WD in the vehicle group, which was entirely blocked by apelin-13 treatment. ACE protein expression in the kidney cortex but not medulla was also increased in WD mice and blocked in apelin-13-treated mice.

### Apelin-13 Counteracts AVP-Induced AQP2 Expression by Inhibiting cAMP/PKA/sPRR Signaling in Primary IMCD Cells

**Figure 5:**
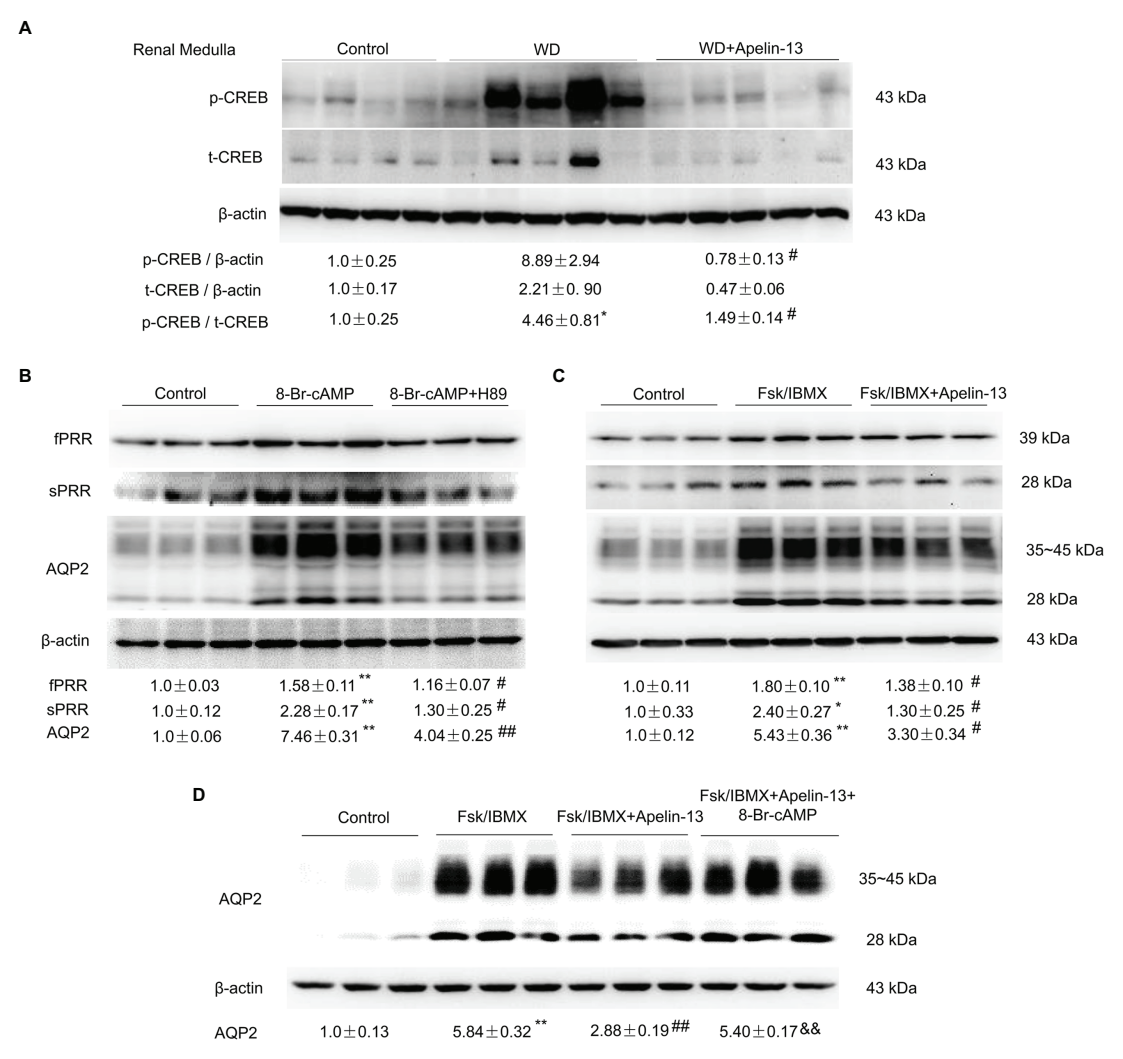
Apelin-13 regulates PRR and AQP2 *via* the cyclic adenosine monophosphate (cAMP)-protein kinase A (PKA) signaling pathway. **(A)** Representative immunoblotting and densitometric analysis of kidney medullary p-CREB (Ser133) and CREB protein expression in the WD mice, β-actin as an internal control for equal loading of the samples. *n* = 4–5 per group. Data are mean ± SEM. ^*^p < 0.05 vs. Control, ^#^*p* < 0.05 vs. WD. **(B)** Effect of cAMP/PKA pathway on PRR and AQP2 expression in the primary rat inner medullary collecting duct (IMCD) cells. Cells were pretreated with H89 and then treated with 100 μM 8-Br-cAMP for 24 h. The expression of PRR and AQP2 protein was analyzed by immunoblotting and normalized by β-actin. *n* = 3 per group, repeat three times. Data are mean ± SEM. ^**^p < 0.01 vs. Control; ^#^*p* < 0.05 and ^##^*p* < 0.01 vs. 8-Br-cAMP. **(C)** Effect of apelin-13 on PRR and AQP2 expression in forskolin (FSK)/3-Isobutyl-1-methylxanthine (IBMX)-treated IMCD cells. Cells were pretreated with 100 nM apelin-13 and then treated with FSK (10 μM)/IBMX (100 μM) for 24 h. The expression of PRR and AQP2 protein was analyzed by immunoblotting and normalized by β-actin. *n* = 3 per group, repeat three times. Data are mean ± SEM. ^*^p < 0.05 and ^**^p < 0.01 vs. Control; ^#^*p* < 0.05 vs. FSK/IBMX. **(D)** Effect of cAMP on AQP2 protein expression in FSK/IBMX + apelin-13-treated IMCD cells. Cells were pretreated with 100 nM apelin-13, or in combination with 100 μM 8-Br-cAMP, then treated with FSK (10 μM)/IBMX (100 μM) for 24 h. The expression of AQP2 protein was analyzed by immunoblotting and normalized by β-actin. *n* = 3 per group. Data are mean ± SEM. ^**^p < 0.01 vs. Control; ^##^*p* < 0.01 vs. FSK/IBMX, ^&&^*p* < 0.01 vs. FSK/IBMX + apelin-13.

**Figure 6:**
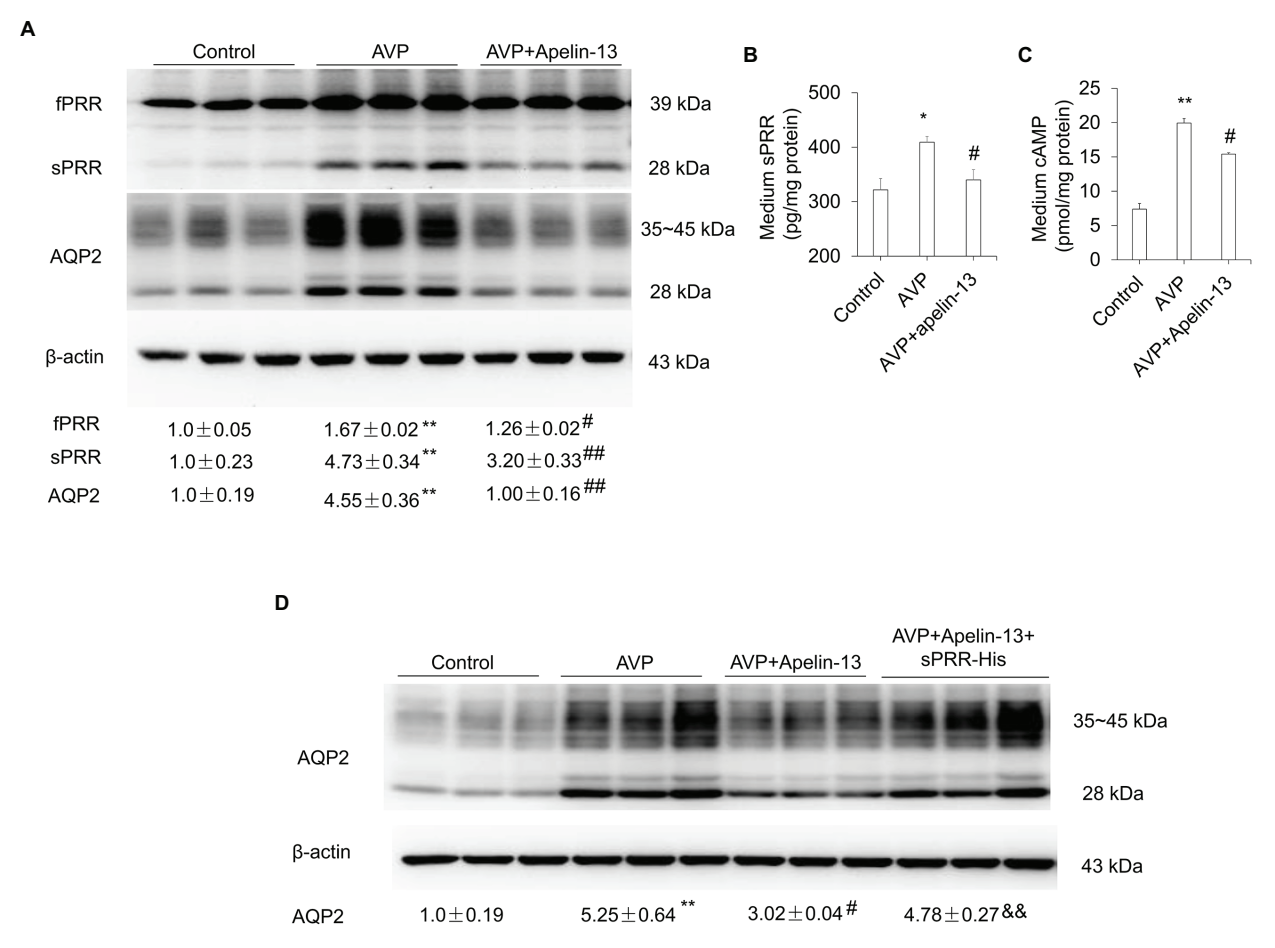
Apelin-13 reduces AQP2 expression through inhibition of sPRR generation in primary IMCD cells. **(A)** Effect of apelin-13 on PRR expression in antidiuretic action of vasopressin (AVP)-treated IMCD cells. Cells were pretreated with 100 nM apelin-13 and then treated with 10 nM AVP for 24 h. The expression of PRR and AQP2 protein was analyzed by immunoblotting and normalized by β-actin. **(B)** Medium sPRR levels. **(C)** Medium cAMP levels. *n* = 3 per group, repeat three times. Data are mean ± SEM. ^*^p < 0.05 and ^**^p < 0.01 vs. Control; ^#^*p* < 0.05 and ^##^*p* < 0.01 vs. AVP. **(D)** Role of sPRR on AQP2 protein expression in AVP + apelin-13-treated IMCD cells. Cells were pretreated with 100 nM apelin-13, or in combination with 10 nM sPRR-His, then treated with 10 nM AVP for 24 h. The expression of AQP2 protein was analyzed by immunoblotting and normalized by β-actin. *n* = 3 per group, repeat three times. Data are mean ± SEM. ^**^p < 0.01 vs. Control; ^#^*p* < 0.05 vs. AVP, ^&&^*p* < 0.01 vs. AVP + apelin-13.

It is well-known that the cAMP/PKA/CREB pathway contributes to the stimulation of AQP2 expression and trafficking (Nielsen et al., 2002). We found that apelin-13 treatment markedly blocked WD-induced phosphorylation of CREB in the kidney medulla (Figure 5A). To further investigate whether apelin inhibits AQP2 expression *via* the inhibition of cAMP/PKA pathway, the primary IMCD cells were incubated with 8-Br-cAMP in the presence or absence of PKA inhibitor H89 or a cocktail of the adenylate cyclase stimulator FSK and the phosphodiesterase inhibitor IBMX or AVP in the presence or absence of apelin-13. H89 partially blocked 8-Br-cAMP-induced upregulation of PRR/sPRR and AQP2 protein expression (Figure 5B). Stimulation with FSK/IBMX triggered a significant increase in the expression of PRR/sPRR and AQP2 protein which was abolished by apelin-13 (Figure 5C). We also observed that 8-Br-cAMP could reverse the inhibitory effect of apelin-13 on FSK/IBMX-induced AQP2 expression (Figure 5D), suggesting that apelin inhibits AQP2 *via* cAMP/PKA pathway. In addition, apelin-13 inhibited AVP-induced increase of PRR/sPRR and AQP2 protein expression as assessed by immunoblotting (Figure 6A), accompanied with decreased medium sPRR levels (Figure 6B) and cAMP levels (Figure 6C) as determined by ELISA. Interesting, exogenous sPRR protein incubation partially reversed the inhibitory effect of apelin-13 on AQP2 protein expression in AVP-treated IMCD cells (Figure 6D). These results indicate that apelin may regulate AQP2 expression *via* the inhibition of the cAMP/PKA/sPRR pathway.

### Apelin-13 Blocks AVP-Induced AQP2 Expression by Inhibiting cAMP/β-Catenin/sPRR Signaling in Primary IMCD Cells

**Figure 7:**
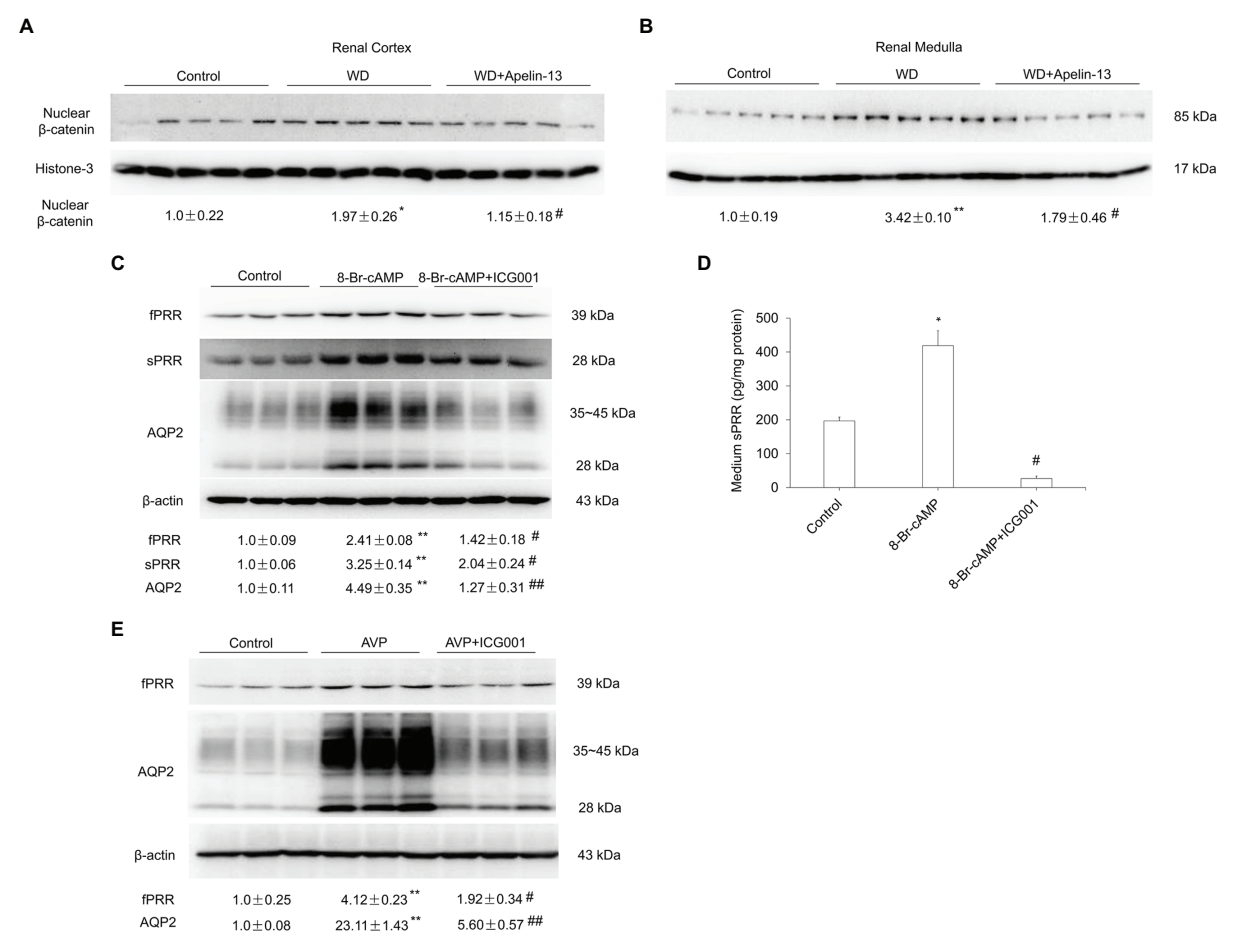
Apelin regulates renal AQP2 expression *via* the cAMP/β-catenin/sPRR signaling pathway. **(A,B)** Representative immunoblotting and densitometric analysis of nuclear β-catenin protein expression in the renal cortex **(A)** and medulla **(B)** in WD mice. The expression of AQP2 protein was analyzed by immunoblotting and normalized by Histone-3 as an internal control for equal loading of the samples. *n* = 5 per group. Data are mean ± SEM. ^*^p < 0.05 and ^**^p < 0.01 vs. control; ^#^*p* < 0.05 vs. WD. **(C,D)** Role of Wnt/β-catenin signaling pathway in response to 8-Br-cAMP in primary rat IMCD cells. Cells were pretreated with ICG001 and then treated with 100 μM 8-Br-cAMP for 24 h. **(C)** The expression of PRR and AQP2 protein was analyzed by immunoblotting and normalized by β-actin. **(D)** Medium sPRR levels were determined. *n* = 3 per group, repeated for three times. Data are mean ± SEM. ^*^p < 0.05 and ^**^p < 0.01 vs. Control; ^#^*p* < 0.05 and ^##^*p* < 0.01 vs. 8-Br-cAMP. **(E)** Role of Wnt/β-catenin signaling pathway in response to AVP in primary rat IMCD cells. Cells were pretreated with ICG001 and then treated with 10 nM AVP for 24 h. The expression of PRR and AQP2 protein was analyzed by immunoblotting and normalized by β-actin. *n* = 3 per group, repeated for three times. Data are mean ± SEM. ^**^p < 0.01 vs. Control; ^#^*p* < 0.05 and ^##^*p* < 0.01 vs. AVP.

Increasing evidence has demonstrated that β-catenin signaling plays an important role in fluid homeostasis *via* the regulation of *aqp2* and *V2R* gene transcription (Jung et al., 2015; Ando et al., 2016; Lu et al., 2016; Wang et al., 2019). The abundance of β-catenin protein was increased in the nuclear fraction from both kidney cortex and medulla following WD as shown previously (Lu et al., 2016) and this increase was attenuated in apelin-13-treated mice (Figures 7A,B). ICG001, a specific inhibitor of Wnt/β-catenin signaling pathway, significantly blocked 8-Br-cAMP or AVP-stimulated PRR/sPRR and AQP2 expression in primary IMCD cells (Figures 7C–E). These results indicate that apelin may regulate AQP2 expression *via* the inhibition of cAMP/β-catenin/sPRR pathway.

## Discussion

Apelin (De Mota et al., 2004; Reaux-Le Goazigo et al., 2004; Roberts et al., 2009; Hus-Citharel et al., 2014; Flahault et al., 2017; Boulkeroua et al., 2019) and PRR (Yang, 2017; Yang and Chuanming, 2017) exert opposing roles in regulation of fluid homeostasis. Activation of PRR *via* S1P-derived sPRR upregulates renal AQP2 expression and promotes urine concentrating capability (Lu et al., 2016; Wang et al., 2019) whereas apelin downregulates AQP2 expression and causes urine concentrating defect (Hus-Citharel et al., 2014; Boulkeroua et al., 2019). The present study is the first to report the potential interaction between the two systems in the setting of water hemostasis.

Apelin and APJ were found in various tissues, including the brain, heart, and kidneys. Previous studies showed that systemic apelin and AVP secretion are significantly altered in various water metabolism disorders, including hyponatremia and polyuria-polydipsia syndrome (Flahault et al., 2017). WD or salt-loading increases plasma osmolality, while WL decreases plasma osmolality in humans and rodents. Plasma AVP and apelin work conversely and apelin is regulated to facilitate systemic AVP release and to avoid additional water loss at the kidney level (De Mota et al., 2004; Azizi et al., 2008). Increased plasma osmolality by WD or salt loading simultaneously raised plasma AVP levels and decreased plasma apelin levels (De Mota et al., 2004; Azizi et al., 2008). Conversely, reduced plasma osmolality by WL reduced plasma AVP levels and rapidly increased plasma apelin levels (Azizi et al., 2008). These observations are consistent with plasma osmolality being a major physiologic regulator of plasma apelin levels in humans and rodents. In the present study, we first demonstrate that the mRNA expression of apelinergic system components in the kidney, including *Apela*, *Apln*, and *Aplnr*, are significantly altered following WD or chronic WL.WD significantly downregulated, whereas chronic WL markedly increased, renal *Apela*, *Apln*, and *Aplnr* mRNA expression and 24-h urinary apelin excretion. Therefore, these results indicate that, apart from systemic apelin secretion, renal-derived apelin following osmotic stress may act locally to control body fluid homeostasis.

It is well known that both apelin and ELA exert a diuretic effect in rodents. In particular, intravenous injection of increasing doses of apelin-17 in lactating rats dose-dependently increases diuresis, accompanied with a decrease in AQP2 trafficking in the CD cells and a significant decrease in urine osmolality (Hus-Citharel et al., 2014). Similarly, acute or chronic intravenous injection of ELA potently increased diuresis in male SD rats (Deng et al., 2015). Of note, the apelin response to dehydration is, therefore, opposite to that of AVP (De Mota et al., 2004; Reaux-Le Goazigo et al., 2004). To support this, herein, apelin-13 partially blunted the increase of urine osmolality and further increased plasma osmolality in WD mice, accompanied with downregulation of renal AQP2 expression, as characterized by the downregulated total, membrane, and cytoplasm AQP2 protein abundance as well as AQP2 mRNA levels. These results were consistent with a previous report that apelin-13 antagonized the hydroosmotic effect of AVP on regulating AQP2 mRNA and protein expression Therefore, these results indicate that apelin exerts an opposite physiological action to mitigate the antidiuretic activity of AVP, at least in part, by a direct action on AQP2 expression in the CDs.

Sodium reabsorption *via* NKCC2 in the TAL plays a significant role in generation of medullary osmotic gradient, an essential component of urine concentrating capability. Thus, NKCC2 has been expected to be activated by WD (Knepper et al., 1999). However, previous studies in rats produced variable results with water restriction but not WD stimulating renal medullary NKCC2 expression, and long-term AVP infusion increasing NKCC2 expression in Brattleboro rats but not normal SD rats (Ecelbarger et al., 1996, 2001). Our data show that 48-h WD markedly increased renal medullary NKCC2 mRNA and protein expression in C57BL/6 J mice. One possibility is that the species difference contributes to these inconsistent results during WD, the presence and activation of inhibitory factors, such as bradykinin (Itoh et al., 2014), SPAK isoforms (Grimm et al., 2012; Park et al., 2013), and PGE2 (Itoh et al., 2014), in the normal SD rats but not mice during WD or AVP infusion. What’s more, dehydration is known to increase plasma AVP concentrations and plasma and urine osmolality. Several studies showed that hyperosmolality, as well as chronic salt loading, increase plasma and urine osmolality, which markedly increased NKCC2 expression/activity in medullary TAL (Ecelbarger et al., 1996; Schiessl et al., 2013; Itoh et al., 2014). These data suggest that dehydration may increase NKCC2 expression in medullary TAL *via* increased plasma AVP level after elevation of plasma and urine osmolality. In our current study, the administration of apelin-13 attenuated the induction of NKCC2 expression, and partially blunted the increase of urine osmolality, but further increased plasma osmolality. These results indicate that hyperosmolality of urine leads to the increase of NKCC2 expression in medullary TAL, Therefore, these results suggest that apelin influences the expression of NKCC2 in WD mice, thus regulates the overall urine concentrating capability.

Increasing evidence has demonstrated that apelin/ELA-induced activation of APJ signaling may serve as a negative regulator of on the RAS (Chun et al., 2008; Sato et al., 2013, 2017, 2019; Siddiquee et al., 2013; Zhang et al., 2017). More importantly, emerging evidence has demonstrated that PRR/sPRR and ELA/Apelin have opposite functions in the regulation of renal handling of Na^+^ and blood pressure and urine concentrating ability (Chun et al., 2008; Deng et al., 2015; Lu et al., 2016; Wang et al., 2016; Sato et al., 2017; Schreiber et al., 2017; Yang and Chuanming, 2017; Xu et al., 2020). Herein, we found that WD-induced upregulation of sPRR and renin expression in the kidney, as well as AVP-induced sPRR expression in primary IMCD cells, was significantly blocked by the administration of apelin-13. It seems reasonable to speculate that apelin may directly inhibit sPRR-mediated activation of intrarenal RAS, and subsequently suppress renal AQP2 expression and water reabsorption in the CDs. To support this, we found that exogenous sPRR-His treatment partially reversed apelin-13-downregulated-AQP2 protein expression in AVP-treated IMCD cells. We recently reported that PRR/sPRR inhibited apelin expression (Xu et al., 2020). In the renal cell-specific PRR knockout mice, including CD PRR KO and nephron PRR KO mice, renal medulla *Apela* and *Apln* mRNA levels, and 24-h urinary ELA and apelin excretion were all increased (Xu et al., 2020). These results were consistent with our *in vitro* results that PRR knockdown by PRR siRNA promoted whereas exogenous sPRR-His treatment inhibited *Apela* and *Apln* mRNA expression in cultured IMCD3 cells (Xu et al., 2020). Therefore, these results support the antagonistic interaction between ELA/apelin and PRR/sPRR in the distal nephron that appears to exert a significant impact on urine concentrating ability.

It is well established that, AVP, upon binding to its V2R, activates the cAMP/PKA/CREB pathway to enhance AQP2 gene transcription (Montminy, 1997; Hasler et al., 2009), and promotes AQP2 trafficking *via* Akt/AS160 and cAMP/PKA pathways (Jung and Tae-Hwan, 2010; Moeller et al., 2011). Along this line, our data showed that 8-Br-cAMP-induced upregulation of AQP2 and sPRR protein expression was significantly abolished by the administration of PKA inhibitor H89 in primary cultured IMCD cells. Apelin, binding to APJ, activates the Gi signaling pathway, including inhibition of adenylate cyclase and stimulation of MAP kinase phosphorylation (Deng et al., 2015). Both apelin-17 and apelin-13 have been reported to reduce AVP-induced cAMP production (Hus-Citharel et al., 2014; Boulkeroua et al., 2019). In the present study, apelin-13 dramatically abolished CREB phosphorylation in WD mice, and FSK/IBMX-induced sPRR and AQP2 protein expression in primary IMCD cells. We also found that a significant decrease in medium cAMP levels was observed when IMCD cells were treated with a combination of AVP and apelin-13, accompanied with a reduction of sPRR and AQP2 protein expression. These results suggest that inhibition of sPRR expression by apelin may be due to suppressed cAMP/PKA signaling, leading to reduced AQP2 expression in the CDs. Overall, these results indicate that apelin inhibits cAMP/PKA/sPRR pathway to suppress AQP2 expression in the CDs.

It is well recognized that β-catenin plays a key role in AVP-stimulated AQP2 expression in the renal CDs (Jung et al., 2015; Ando et al., 2016; Lu et al., 2016; Wang et al., 2019). In the present study, our data showed that Wnt/β-catenin signaling inhibitor, ICG001, significantly blocked the upregulation of PRR/sPRR and AQP2 expression in 8-Br-cAMP or AVP-treated IMCD cells. Renal β-catenin signaling is activated, as characterized by the increase of nuclear β-catenin abundance in both cortex and medulla following WD (Lu et al., 2016), which was blocked by apelin-13, accompanied with downregulation of renal AQP2 expression in WD mice. Thus, these results suggest the inhibition of β-catenin signaling, blocks the 8-Br-cAMP-induced sPRR production, and causes decreased AQP2 expression and impaired urine-concentrating capability. Therefore, these results suggest that apelin regulates AQP2 expression and fluid homeostasis by inhibiting cAMP/β-catenin/sPRR signaling pathway.

In summary, the present study reports for the first time that sPRR is downstream of cAMP/PKA to mediate AVP-induced AQP2 expression and serves as a molecular target of apelin-13 to counteract antidiuretic action of AVP. This study provides a novel insight into the mechanisms responsible for the diuretic action of apelin in the distal nephron.

## Data Availability Statement

The original contributions presented in the study are included in the article/supplementary material; further inquiries can be directed to the corresponding author.

## Ethics Statement

The animal study was reviewed and approved by Animal Care and Use Committee at Sun Yat-sen University, China.

## Author Contributions

TY: conceived and designed research, edited and revised manuscript, and approved final version of manuscript. YC: performed experiments, analyzed data, interpreted results of experiments, prepared figures, and drafted manuscript. CX: performed experiments, analyzed data, and interpreted results of experiments. JH: performed experiments, analyzed data, and prepared figures. QQ and MD: performed experiments and analyzed data. SM: performed experiments. All authors contributed to the article and approved the submitted version.

### Conflict of Interest

The authors declare that the research was conducted in the absence of any commercial or financial relationships that could be construed as a potential conflict of interest.
